# Sarah Elizabeth Stewart

**DOI:** 10.3201/eid2005.131876

**Published:** 2014-05

**Authors:** Carl Fulghieri, Sharon Bloom

**Affiliations:** Carrboro High School, Carrboro, North Carolina, USA (C. Fulghieri);; Centers for Disease Control and Prevention, Atlanta, Georgia, USA (S. Bloom)

**Keywords:** polyomavirus, viruses, history of medicine, oncogenic virus, cancer, Sarah Elizabeth Stewart, virologist, Bernice Eddy

This is a photograph of Sarah Elizabeth Stewart, PhD, MD (1905–1976) (Figure), whose discoveries involving the murine polyomavirus with Bernice Eddy, PhD, propelled the then-reluctant field of oncology to pursue viral etiologies of cancer. Sarah Stewart was born on August 16, 1905, in Jalisco, Mexico, to an American engineer and Mexican mother. After moving with her family back to the United States at age 5, Stewart remained fluent in Spanish. In 1927, she graduated from New Mexico State University and in 1930, she earned an MS in Microbiology at the University of Massachusetts, Amherst. She worked at the National Institutes of Health (NIH) during 1935–1944, publishing 7 papers on anaerobic bacteria and completing a PhD from the University of Chicago in 1939.

**Figure:**
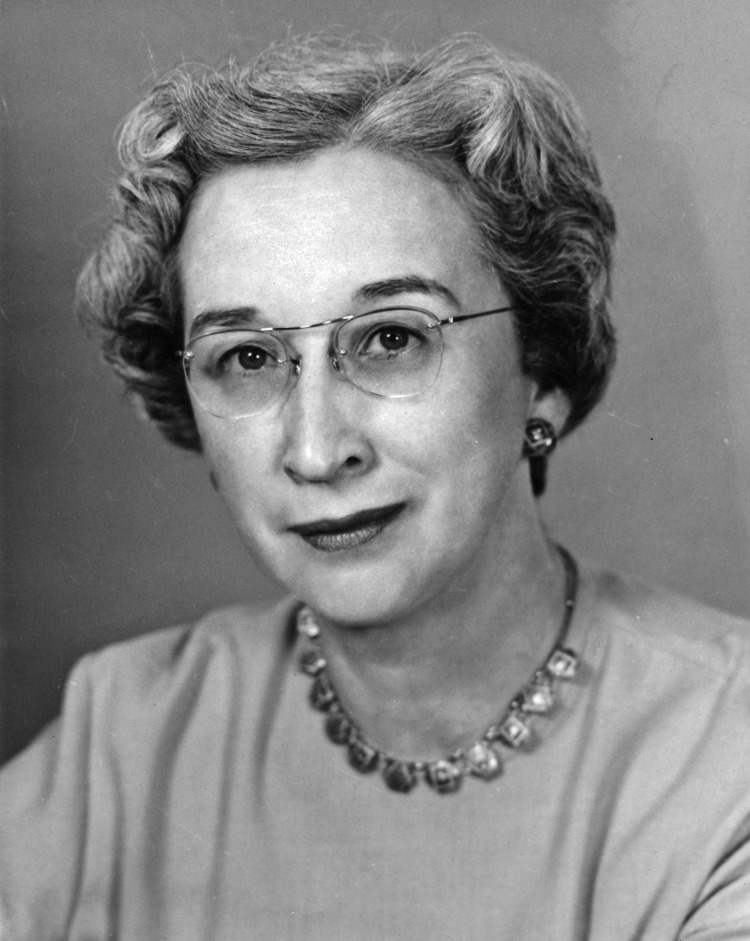
Sarah Elizabeth Stewart (1905–1976). Source: Hobson-Huntsinger University Archives at New Mexico State University.

In 1944, when Stewart requested support to study the link between animal tumors and viruses, the directors of the NIH Laboratory of Microbiology and the National Cancer Institute (NCI) refused on the grounds that the proposal seemed dubious and that she lacked appropriate qualifications. Rather than give up her passion to study viruses and cancer, Stewart resigned and became a bacteriology instructor at Georgetown University School of Medicine; there she audited classes, and after women were permitted to enroll, Stewart became their first woman graduate at age 39. After completing an internship in 1951, Stewart returned to NCI to launch her viral oncology research career.

To understand Stewart’s role in catalyzing viral oncology research, it is necessary to recognize that until the 1950s, scientists dismissed the idea that viruses could cause cancer. It took many decades before the seminal contributions of several virologists studying cancers were appreciated, such as Peyton Rous’ 1911 discovery of the Rous sarcoma virus (which caused tumors in chickens), and discoveries of Richard Shope (rabbit fibroma) and John Bittner (mouse mammary carcinoma) in the 1930s. In 1951, Ludwig Gross described the transmission of leukemia in newborn mice by using a cell-free extract; in 1953, he reported parotid tumors in these mice. Even after Stewart confirmed Gross’s findings in 1953, the scientific community still did not acknowledge viral causes of mammalian cancer. Only after Stewart fulfilled Koch’s postulates in 1957, with the assistance of Dr. Bernice Eddy, did oncologists pay heed to viruses.

Dr. Eddy had also trained as a PhD bacteriologist at the University of Chicago and since 1937, worked at the NIH Biologics Control Laboratories, in Bethesda, Maryland. In 1954, Eddy had been sidelined for whistleblowing about the presence of live virus in Jonas Salk’s inactivated polio vaccine (the infamous Cutter incident). So in 1956, when Stewart approached Eddy for assistance growing the agent causing parotid tumors in mice, Eddy readily agreed and the 2 women rapidly worked out the characteristics of the agent that was not referred to as a virus in their publications until 1959. Together they showed that the virus produced 20 types of mouse tumors and could cause tumors in other small mammals. At Eddy’s suggestion, the virus was dubbed polyoma, which means many tumors, and they named it the SE (Stewart–Eddy) polyomavirus. They also demonstrated that the virus causes cell necrosis and proliferation in cell culture, that it is highly antigenic, and that it leads to formation of specific antibodies in infected animals whether or not tumors develop.

The results of their collaboration were picked up by a 1959 Time Magazine cover story, citing John Heller, then the NCI director, “the hottest thing in cancer research is research on viruses as possible causes of cancer.” Alan Rabson, a prominent member of the NCI Laboratory of Pathology, stated, “The whole place just exploded after Sarah found polyoma.”

Stewart became medical director of the NCI Laboratory of Oncology and spent the remainder of her life researching several oncogenic viruses (e.g., Epstein-Barr virus). As a US Public Health Service Commissioned Officer, her scientific contributions to the study of viral etiologies of cancer earned her the Federal Women’s Award, presented by President Lyndon Johnson in 1965. In 1960, Eddy again found herself in hot water, this time for reporting her discovery of an oncogenic simian virus (SV40) in polio vaccine prepared from monkey kidneys. Stewart retired from the Public Health Service in 1970 to become a full professor of pathology at Georgetown University. She died of stomach cancer in 1976. Bernice Eddy described her as “a forceful individual who did not let anything stand in [her] way if she could help it.” Despite sex discrimination and a period in which several laboratory directors disparaged her wish to study oncogenic viruses, Stewart persisted with such enthusiasm that she managed to break through as one of the most influential scientists and cancer researchers of her time.
